# PET Imaging a MPTP-Induced Mouse Model of Parkinson**’**s Disease Using the Fluoropropyl-Dihydrotetrabenazine Analog [^18^F]-DTBZ (AV-133)

**DOI:** 10.1371/journal.pone.0039041

**Published:** 2012-06-18

**Authors:** James S. Toomey, Shilpa Bhatia, La’Wanda T. Moon, Elysse A. Orchard, Kerrie H. Tainter, Stephen J. Lokitz, Tracee Terry, J. Michael Mathis, Andrew D. Penman

**Affiliations:** 1 Southern Research Institute, Birmingham, Alabama, United States of America; 2 Department of Animal Resources, Louisiana State University Health Sciences Center, Shreveport, Louisiana, United States of America; 3 Department of Cellular Biology and Anatomy, Louisiana State University Health Sciences Center, Shreveport, Louisiana, United States of America; 4 Department of Pharmacology, Toxicology, and Neuroscience, Louisiana State University Health Sciences Center, Shreveport, Louisiana, United States of America; 5 Gene Therapy Program, Louisiana State University Health Sciences Center, Shreveport, Louisiana, United States of America; 6 Feist-Weiller Cancer Center, Louisiana State University Health Sciences Center, Shreveport, Louisiana, United States of America; 7 The Biomedical Research Institute of Northwest Louisiana, Shreveport, Louisiana, United States of America; The University of Chicago, United States of America

## Abstract

Parkinson’s disease (PD) is characterized by the loss of dopamine-producing neurons in the nigrostriatal system. Numerous researchers in the past have attempted to track the progression of dopaminergic depletion in PD. We applied a quantitative non-invasive PET imaging technique to follow this degeneration process in an MPTP-induced mouse model of PD. The VMAT2 ligand ^18^F-DTBZ (AV-133) was used as a radioactive tracer in our imaging experiments to monitor the changes of the dopaminergic system. Intraperitoneal administrations of MPTP (a neurotoxin) were delivered to mice at regular intervals to induce lesions consistent with PD. Our results indicate a significant decline in the levels of striatal dopamine and its metabolites (DOPAC and HVA) following MPTP treatment as determined by HPLC method. Images obtained by positron emission tomography revealed uptake of ^18^F-DTBZ analog in the mouse striatum. However, reduction in radioligand binding was evident in the striatum of MPTP lesioned animals as compared with the control group. Immunohistochemical analysis further confirmed PET imaging results and indicated the progressive loss of dopaminergic neurons in treated animals compared with the control counterparts. In conclusion, our findings suggest that MPTP induced PD in mouse model is appropriate to follow the degeneration of dopaminergic system and that ^18^F-DTBZ analog is a potentially sensitive radiotracer that can used to diagnose changes associated with PD by PET imaging modality.

## Introduction

According to the Parkinson’s disease Foundation, Parkinson’s disease (PD) is estimated to currently affect as many as 1 million Americans; about 50 to 60,000 Americans are diagnosed with the disease every year. The causes of PD are still unknown; but genetics may be a contributing factor and environmental toxins may also play a role [1]. Investigators have used experimental models of PD to provide insights into the mechanisms responsible for the loss of dopaminergic neurons in PD [2]. The chemically induced mouse model of PD using 1-methyl-4-phenyl-1,2,3,6-tetrahydropyridine (MPTP) has been widely used in developing and evaluating new treatments of PD [3]. MPTP is capable of causing clinical signs found in PD by producing a reliable and reproducible lesion of the substantia nigra.

Non-invasive imaging studies using positron emission tomography (PET) have been useful for monitoring the loss of dopaminergic neurons in PD patients and animal models with the radiotracer 6-[^18^F]-fluoro-L-DOPA (^18^F-DOPA). However, ^18^F-DOPA is rapidly metabolized in the periphery following intravenous injection, limiting its utility [4]. Thus, alternative radiotracers have been investigated. Dihydrotetrabenazine (DTBZ) analogs have been investigated as radiotracers for the vesicular monoamine transporter (VMAT2) [5], expressed in the central nervous system (CNS) [6], as well as in pancreatic beta cells [7], [8]. VMAT2 plays a role in vesicular packaging and storage of monoamine neurotransmitters in the synapses of the brain, where it is responsible for the movement of the monoamines (dopamine, serotonin, and norepinephrine) from the cytosol into the vesicular lumen [9]. VMAT2 is highly expressed in the striatum, hypothalamus, substantia nigra, and hippocampus, with lower levels in the cerebellum and occipital cortex [10], [11]. Decreases of VMAT2 levels have been associated with PD [12], [13].

In models of PD, ^3^H-radiolabeled VMAT2 ligand analogs have demonstrated loss of binding and have provided an accurate assessment of monoamine neuronal loss. Non-invasive imaging of CNS receptors in particular requires high specific activity ligands. Using the positron emitter^ 11^C, the [^11^C](+)-DTBZ radiotracer has been useful for imaging changes in numbers of dopaminergic neurons by PET [5]. However, the relatively short half-life of ^11^C (20 min) and manufacturing constraints has limited this radiotracer from widespread clinical use. More recently, an ^18^F-radiolabeled DTBZ analog has been developed, providing a radiotracer with a longer half-life (110 min) and the potential for clinical use [6], [14]. While this analog is currently being evaluated in clinical trials [15] and in a rat model of PD [16], the capability of this radiotracer has not been examined using a mouse model *in vivo*. The purpose of this study was to evaluate the MPTP mouse model of PD using ^18^F-DTBZ specific for VMAT2 to follow loss of dopaminergic neurons. This non-invasive imaging approach can add value to the MPTP mouse model and can assist in the development of new treatments for PD.

## Materials and Methods

### Ethics Statement

All animals used in this study received humane care based on guidelines set by the American Veterinary Association as well as in accordance with the *Guide for the Care and Use of Laboratory Animals* (Institute for Laboratory Animal Research, Washington, DC). The experimental protocols involving live animals were reviewed and approved by the Institutional Animal Care and Use Committee of LSU Health Sciences Center at Shreveport (protocol P-10-028). All efforts were made to minimize animal suffering, to reduce the number of animals used and to utilize alternatives to in vivo techniques, if available.

### Animals

Male C57BL/6NCrl mice between 8 and 10 weeks of age were obtained from the Jackson Laboratory (Bar Harbor, ME).

### Dosing Procedure

At the start of each dosing, MPTP obtained from Sigma Aldrich (St. Louis, MO) was reconstituted with sterile saline solution to a concentration of 5 mg/ml. On Day 1, mice in one of three dose groups were given four IP doses (2 hours apart) of saline, MPTP at 15 mg/kg or MPTP at 17 mg/kg and kept warm throughout the dosing period. Each mouse was euthanized by CO_2_ asphyxiation on Day 15. For each animal, the brain was harvested and the striatum was extracted, snap frozen with liquid N_2,_ and stored at −80°C.

### Tissue Extraction

Brain sections were homogenized in 200 – 750 µL of 0.1 M trichloroacetic acid (TCA), which contained 10^−2^ M sodium acetate, 10^−4^ M EDTA and 10.5% methanol (pH 3.8) using a tissue dismembrator (Fisher Scientific; Pittsburgh, PA). Samples were spun in a microcentrifuge at 10,000×g for 20 minutes and analyzed for biogenic monoamines and/or amino acids.

### Analysis of Biogenic Amines

Samples of brain striatum were processed and analyzed at the Neurochemistry Core lab at Vanderbilt University (Nashville TN). Biogenic amines were determined based on a specific HPLC assay as previously described [17] utilizing an Antec Decade II (oxidation: 0.5) electrochemical detector operated at 33°C. Samples of the supernatant (20 µL) were injected using a Water 717+ autosampler onto a Phenomenex Nucleosil (5 μ, 100 A) C18 HPLC column (150×4.60 mm). Biogenic amines were eluted with a mobile phase consisting of 89.5% 0.1 M TCA, 10^−2^ M sodium acetate, 10^−4^ M EDTA, and 10.5% methanol (pH 3.8). Solvent was delivered at 0.8 mL/min using a Waters 515 HPLC pump. Using this HPLC solvent, the following biogenic amines eluted in the following order: noradrenaline, adrenaline, DOPAC, Dopamine, 5-HIAA, HVA, 5-HT, and 3-MT. Concentrations are expressed as nanograms per milligram of total protein.

### Protein Determination

Total protein concentration of the brain extracts was determined using a BCA Protein Assay Kit purchased from Pierce Chemical Company (Rockford, IL). The frozen pellets were allowed to thaw and reconstituted in a volume of 0.5 N HCl equal to that previously used for tissue homogenization. A 100 µL aliquot of this solution was combined with 2 mL of color reagent and allowed to develop for 2 hours. A BSA standard curve was run at the same time spanning the concentration range of 20 – 2000 µg/mL. Absorbances of standards and samples were measured at 562 nm.

### Immunohistochemical Analysis

Brain samples were stained for detection of tyrosine hydroxylase (TH) to visualize the effect of MPTP treatment. Samples of brain striatum were post-fixed in 10% buffered formalin (48 h, 4°C). The fixed tissue samples were dehydrated with gradated alcohols, cleared with xylene, and infiltrated with paraffin overnight. Paraffin-embedded tissue samples were then cut into 5 µm-thick sections, which were mounted on slides, deparaffinized with xylene, and rehydrated in gradated alcohols prior to staining. Sections were processed in a 10 mm citrate buffer for enhancement of antigen retrieval. After blocking with 10% donkey serum for 1 h, an anti-TH antibody (Sigma) was applied to the sections for overnight incubation at 4°C. For immunofluorescence staining, a Texas Red conjugated secondary antibody was used for fluorescence detection. For immunohistochemistry staining, a horseradish peroxidase conjugated secondary antibody was used followed by 3,3′ diaminobenzidine processing.

### PET Imaging

The [^18^F]-DTBZ radiotracer (AV133) was prepared from aqueous [^18^F]-fluoride as previously described [18]. Radiochemical purity and specific activity of each synthesis were determined by analytical HPLC. PET studies were performed using a FLEX Triumph™ multi-modality pre-clinical platform (Gamma-Medica Ideas, Northridge, CA) combining three different imaging modalities (PET, SPECT, and CT) in a single platform. In brief, the system features 2×2×12 mm^3^ LYSO and LGSO crystals assembled in phoswich pairs, read out by Avalanche PhotoDiode (APD) detectors, and arranged for a 7.5 cm transaxial field-of-view. For the imaging studies, the mice were injected in the tail vein with 300 µCi of [^18^F]-DTBZ. After 60 minutes, the animals were anesthetized with isoflurane and image data was acquired for 15 minutes. PET images were reconstructed by using an iterative reconstruction technique. The reconstructed pixel size was 0.25×0.25×1.175 mm, which produces voxels of 0.07 mm^3^.

### CT Imaging

The purpose of the CT imaging was alignment of the PET data for registration to a brain atlas [19]. CT images were acquired immediately after PET imaging while the animals were still under anesthesia. CT images were acquired with 80 kVp voltage with optimal current, for 256 projections over 360 degrees. Reconstructed images were either 512×512×512 voxels with a voxel size of 0.1 mm^3^.

### Image Analysis

Initially, one CT scan from a normal control animal was manually reoriented so that bregma and lambda were in the same slice plane and vertically colinear, and the intra-aural canals were in the same plane and horizontally collinear. This position is easily related to atlas coordinates, which commonly reference distances from bregma, the midline, and the intra-aural line [19]. The CT for each subsequent animal was then registered to the first CT using the normalized mutual information algorithm available in SPM2, a statistical image processing utility developed for Matlab (Mathworks Inc., Natick, MA). Finally, PET images were registered to their corresponding CT and resliced in the new orientation having cubic voxel dimensions of 0.2 mm and an origin located at the bregma. Images were then cropped to include only the skull and brain and intensity normalized so that the minimum voxel value was 0 and the maximum voxel value was 1. The methods used for orienting the images were previously developed for use with imaging PD models in rats [20]. Regions of interest for the striatum were identified in the PET images of normal control animals in eleven slices of the image centered on the slice containing bregma, and individual voxels were measured within a circle of radius 10 voxels for both left and right striatum. The total number of cerebellar measurements for each image was 1246 voxels (178 voxels/slice). For comparison with striatum values, regions of the cerebellum were also measured in eleven image slices, using circular regions of 10 voxels radius. The total number of cerebellar measurements for each image was 979 voxels (89 voxels/slice). For both striatal and cerebellar measurements, the mean voxel values for each slice were first determined, and then the means and standard deviations of all slices from each image were obtained. The groups of striatal and cerebellar slices from each animal were subsequently compared using one-way ANOVA followed by pair-wise Tukey’s post-hoc test. The statistics were calculated by the GraphPad InStat version 3.2 software (GraphPad Software, Inc., La Jolla, CA). A P value less than 0.05 was considered statistically significant.

## Results

To implement a mouse model of PD using the neurotoxin MPTP safely and successfully, it is critical to be consistent with MPTP injection route, regimen and mice including strain, vendor, gender, age, and body weight [21]. All of these factors have a significant impact on the sensitivity and reproducibility of the lesion caused by MPTP administration and the degree of dopaminergic neurodegeneration. For these studies, we used C57BL/6NCrl mice between 8 and 10 weeks of age from the Jackson Laboratory. On Day 1, each mouse was administered four IP doses (approximately 2 hours apart) of saline (Group 1) or MPTP (Groups 2 and 3). Animals in Group 2 were given 15 mg/kg doses and animals in Group 3 were given 17 mg/kg doses. The animals in Groups 2 and 3 were noted to be hypoactive during the first 24 hours after dosing with MPTP. These clinical signs resolved and all animals were clinically normal throughout the rest of the study. One Group 3 animal died acutely during the second IP dosing procedure. Due to the circumstances of this animal’s death, it is suspected that the death was secondary to an IP injection error and not directly related to MPTP toxicity. Group 1 (control) animals showed no abnormal clinical signs throughout the course of the study. No treatment-related morbidity or mortality was reported during the study. Body weights for all animals were obtained on days 1 and 15. Body weights were averaged for each group and the percent of body weight gained over the course of the study was determined for each group. Group 1 animals gained 8.4% (2.1±0.7 g) of their day 1 body weight over the course of the study, from 24.4±1.3 g to 26.5±1.6 g. Group 2 animals gained 7.2% (1.7±1.1 g), from 22.9 g±1.3 g to 24.5±2.1 g, and Group 3 animals gained 6.8% (1.6±0.5 g), from 23.0 g±2.1 g to 24.6±2.4 g. The single Group 3 animal that died during dosing on Day 1 was excluded from these calculations. Comparison of weight increase among the three Groups showed no significant difference using a one-way ANOVA analysis.

**Figure 1:**
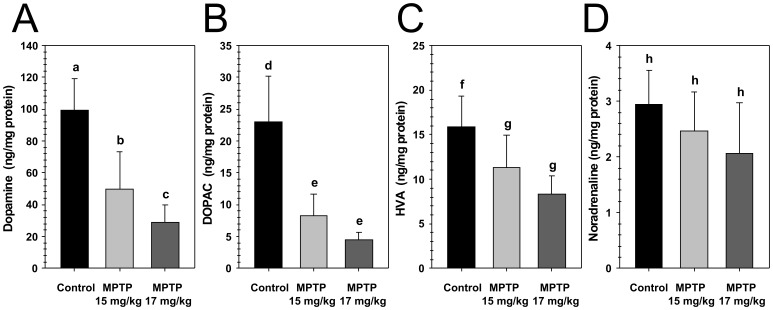
Changes in levels of dopamine and its main metabolites in samples of brain striatum from MPTP treated mice. The degree of dopaminergic neurodegeneration caused by MPTP administration was evaluated by examining the levels of dopamine (A), DOPAC (B), and HVA (C). As a control, the effect of MPTP administration on noradrenaline levels (D) was also evaluated in the same samples. Results of striatum values from control and MPTP treated mice were analyzed by ANOVA followed by Tukey’s post-hoc test. Lowercase letters above the bars indicate the results of that analysis; values that share any letter in common are not significantly different (P ≥ 0.05), and values that do not share any letter in common are significantly different (P < 0.05).

We initially examined the degree of dopaminergic neurodegeneration caused by MPTP administration by evaluating the levels of dopamine and its main metabolites, 3,4-dihydroxyphenylacetic acid (DOPAC) and homovanillic acid (HVA) in samples of brain striatum from control and MPTP treated mice. The results of this analysis are shown in Table 1 and summarized in *Fig. 1*. The control Group 1 (saline only) dosing regimen was used to determine the baseline levels for dopamine, and its two metabolites dihydroxyphenylacetic acid (DOPAC) and homovanillic acid (HVA). For dose Group 2 (15 mg/kg MPTP), there was a 49.8% decrease in dopamine content of the brain striatum relative to the baseline levels (Group 1), from 99.2±20.1 ng/mg protein to 49.8±23.5 ng/mg protein. Likewise, dose Group 2 showed a 63.9% decrease in DOPAC from 23.1±7.1 ng/mg protein to 8.3±3.3 ng/mg protein, and a 29.1% decrease in HVA from 15.9±3.4 ng/mg protein to 11.3±3.7 ng/mg protein. For dose Group 3 (17 mg/kg) there was a 70.9% decrease in dopamine content of the brain striatum relative to the baseline levels (Group 1), from 99.2±20.1 ng/mg protein to 28.9±10.8 ng/mg protein. Likewise, dose Group 3 showed an 81.3% decrease in DOPAC from 23.1±7.1 ng/mg protein to 4.4±1.3 ng/mg protein, and a 47.8% decrease in HVA from 15.9±3.4 ng/mg protein to 8.3±2.1 ng/mg protein. Importantly, the measured decreases in the biogenic amine and its metabolites are consistent with values previously reported for a well-defined working MPTP-induced PD mouse model [22].

**Table 1:** Brain Striatum Levels of Dopamine, Dopamine Metabolites, and Noradrenaline.

Treatment	Animal ID	Dopamine (ng/mg protein)	DOPAC^a^ (ng/mg protein)	HVA^b^ (ng/mg protein)	Noradrenaline (ng/mg protein)
	G1-M1	86.27	26.98	17.03	2.47
	G1-M2	64.44	16.22	8.81	3.86
	G1-M3	88.33	19.25	12.78	2.21
	G1-M4	104.71	40.13	18.55	2.78
**Control**	G1-M5	117.46	25.29	17.96	2.44
	G1-M6	100.14	22.86	16.63	3.17
	G1-M7	80.66	15.75	13.13	2.54
	G1-M8	134.60	23.90	20.54	2.88
	G1-M9	107.06	21.85	16.25	3.17
	G1-M10	108.38	18.37	17.48	4.01
	**Mean±S.D.**	**99.2±20.1**	**23.1±7.1**	**15.9±4.4**	**2.95 ±0.60**
	G1-M11	29.42	7.08	9.73	1.44
	G1-M12	9.18	1.58	3.20	3.18
	G1-M13	45.28	10.36	12.47	2.12
	G1-M14	63.46	12.62	17.05	3.47
**15 mg/kg**	G1-M15	42.42	6.09	10.29	2.28
**MPTP**	G1-M16	54.18	9.86	11.49	3.46
	G1-M17	49.13	9.82	13.61	2.04
	G1-M18	92.92	10.63	14.28	1.69
	G1-M19	74.75	10.21	11.68	2.42
	G1-M20	37.49	5.00	9.16	n.d.^c^
	**Mean±S.D.**	**49.8±23.5**	**8.3±3.3**	**11.3±3.7**	**2.46±0.75**
	G1-M21	25.71	4.46	8.90	1.57
	G1-M22	39.95	5.29	9.10	3.08
	G1-M23	50.83	7.12	12.05	2.28
**17 mg/kg**	G1-M24	22.65	3.57	6.79	1.45
**MPTP**	G1-M25	22.37	3.98	7.14	1.23
	G1-M26	26.94	3.56	7.28	2.11
	G1-M27	30.91	4.44	8.60	1.53
	G1-M28	13.72	2.65	4.81	3.94
	G1-M30	26.83	4.67	10.10	1.37
	**Mean±S.D.**	**28.9±10.8**	**4.4±1.3**	**8.3±2.1**	**2.06±0.91**

a dihydroxyphenylacetic acid metabolite of dopamine.

b homovanillic acid metabolite of dopamine.

c n.d. not determined.

As a control, we examined the effect of MPTP administration on the catecholamines, adrenaline and noradrenaline. The results of this analysis are shown in Table 1 and summarized in *Fig. 1D*. Adrenaline levels were undetectable in tissues from any of the animals examined, with the exception of one control animal (data not shown). However, noradrenaline was detectable, and there were no statistically significant differences between the noradrenaline levels in the brain striatum for any of the groups. This lack of change in the measured noradrenaline suggests the dissections were performed consistently.

**Figure 2:**
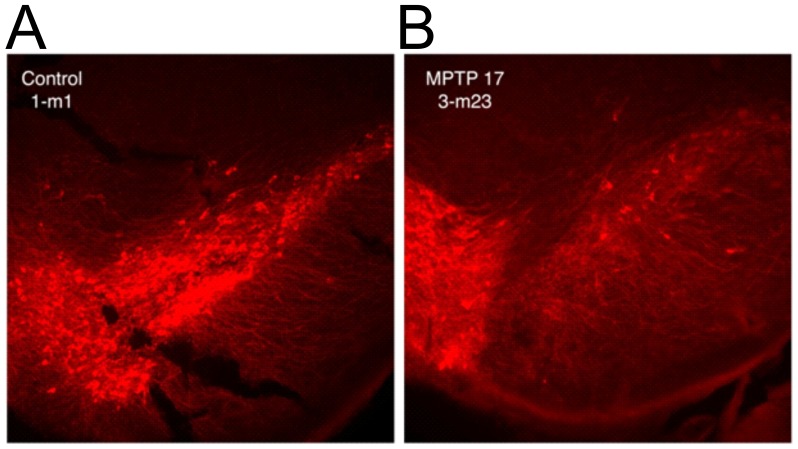
Immunohistochemical visualization of mouse brain striatum samples from control and MPTP treated mice. Tyrosine hydroxylase expression was evaluated by immunofluorescence staining of a brain section from a control untreated mouse (A) or a corresponding brain section from a mouse treated with 17 mg/kg MPTP (B).

To further evaluate the degree of dopaminergic neurodegeneration caused by MPTP administration we performed an immunohistochemical analysis of tyrosine hydroxylase in brain sections from control and MPTP treated mice. Images of a sample from a control (Group 1) animal and one from a high dose (Group 3) animal are shown in *Fig. 2*. In this experiment, immunofluorescence visualization shows a marked decrease in the number of dopamine neurons between the control group and the MPTP-treated groups. The fluorescent staining pattern of tyrosine hydroxylase shows the loss of cells in the striatum of an animal in dose Group 3 (*Fig. 2B*) compared with the striatum of animal from the control group (*Fig. 2A*). In addition, the staining on the left side of each panel shows that the dopamine neurons in the ventral tegmental area (VTA) are less affected by the MPTP than those nigral cells. The VTA neurons are more resistant and as such serve as a good control.

**Figure 3:**
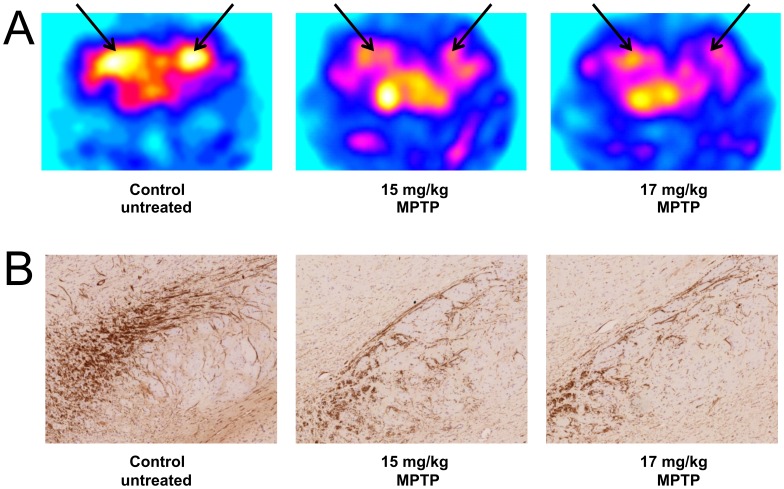
Non-invasive PET imaging of control and MPTP treated mice. A control male C57BL/6NCrl mouse and mice treated with 15 mg/kg MPTP or 17 mg/kg MPTP were imaged by PET using [^18^F]-DTBZ. (A) Shown are coronal brain slices depicting the uptake of [^18^F]-DTBZ corresponding to the anatomical localization of VMAT sites in the striatum. (B) Shown are coronal brain slices from the same animals depicting immunohistochemical visualization of tyrosine hydroxylase activity in dopaminergic neurons.

We performed PET imaging studies of control and MPTP treated mice using the VMAT2 ligand [^18^F]-DTBZ (AV-133). As shown in *Fig. 3A*, a clear decrease in [^18^F]-DTBZ uptake can be seen in the striatum regions of brains from mice treated with 15 mg/kg and 17 mg/kg MPTP compared with a control (untreated) mouse. This loss of uptake suggests that the dopaminergic neurons in the striatum were successfully lesioned and that [^18^F]-DTBZ may be useful for imaging VMAT2 in the mouse brain. To correlate loss of dopaminergic neurons with decreased [^18^F]-DTBZ uptake, we performed an immunohistochemical analysis of tyrosine hydroxylase in brain sections from these mice (*Fig 3B*). In these animals, the staining pattern of tyrosine hydroxylase clearly shows the loss of cells in the striatum of treated with 15 mg/kg and 17 mg/kg MPTP compared with the striatum of the control animal.

**Figure 4:**
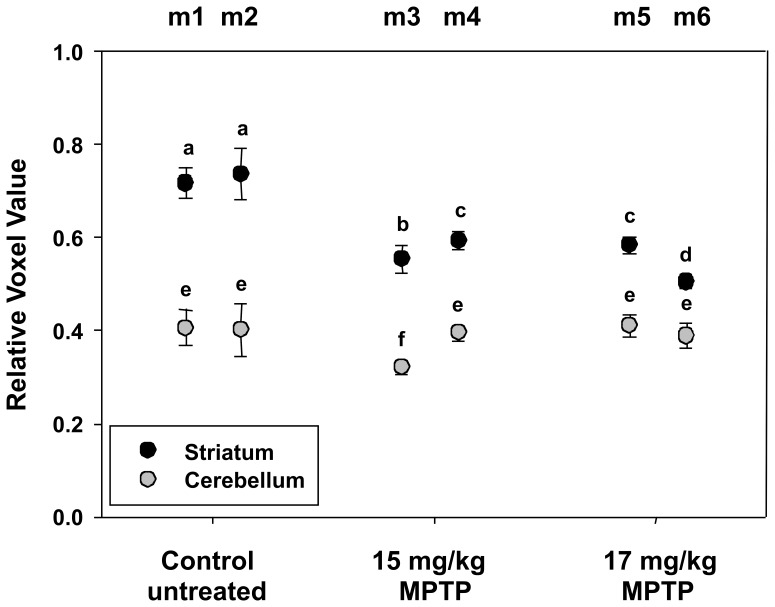
Results from quantification of PET imaging. Control male C57BL/6NCrl mice (m1 and m2) and mice treated with 15 mg/kg MPTP (m3 and m4) or 17 mg/kg MPTP (m5 and m6) were imaged by PET using [18F]-DTBZ. Normalized mean voxel values from regions of interest corresponding to each slice of striatum and cerebellum were obtained, and values shown are means±SD for all image slices from each animal. Results of striatum values from control and MPTP treated mice were analyzed by ANOVA followed by Tukey’s post-hoc test. Lowercase letters above the symbols indicate the results of that analysis; values that share any letter in common are not significantly different (P ≥ 0.05), and values that do not share any letter in common are significantly different (P < 0.05). In a similar fashion, results of cerebellum values from control and MPTP treated mice were analyzed by ANOVA followed by Tukey’s post-hoc test.

Quantitative analysis of the PET images from six animals is shown in *Fig. 4*. In this experiment, regions of interest (ROIs) for the striatum were identified in the PET images of two control animals in seven slices, and individual voxel values were measured within the ROIs for both left and right striatum. Afterwards, the minimum voxel value was normalized to 0 and the maximum voxel value was normalized to 1, and the mean voxel values in each of the seven slices were obtained. The mean and standard deviation of voxel values from all seven slices of each animal were then determined to obtain a relative voxel value. For comparison with striatum values, ROIs of the cerebellum were also measured in eleven image slices using circular regions of radius 10 voxels. As shown in *Fig. 4A*, the MPTP treated mice (m3 – m6) showed significantly lower signal intensities corresponding to decreased [^18^F]-DTBZ striatum uptake, compared with either control mouse m1 (P < 0.0001) or control mouse m2 (P < 0.0001). As a control, the [^18^F]-DTBZ uptake in the cerebellum was also examined. By one-way ANOVA, there was no significant difference when the control mice (m1 and m2) and the MPTP treated mice m4, m5, and m6 were compared (P = 0.639). However, m3 did show a lower background [^18^F]-DTBZ uptake (P < 0.0001) in the cerebellum when compared with any of the other mice by pair-wise Tukey’s post-hoc test.

## Discussion

Parkinson’s disease is a progressive neurological disorder affecting as many as one million Americans. Several animal models have been developed to gain basic understanding about the mechanisms involved in the onset and progression of this progressive neurological disorder [2], [22]. These include toxin, such as MPTP, induced animal models that cause selective depletion of dopamine producing neuronal cells [23]. With the advances in the area of non-invasive imaging, novel radioactive ligands such as [^11^C]- and [^18^F]-DTBZ have been developed that bind to the neurotransmitter receptors with high selectivity and affinity. Radioligands such as these have emerged as potential tools capable of detecting the decline in dopaminergic activity in the affected regions of the brain.

The present study demonstrates the usefulness and efficacy of ^18^F-DTBZ analog as a radioligand for the study of dopaminergic loss in the MPTP induced PD mouse model. The [^18^F]-DTBZ analog is a PET tracer with a relatively longer half-life compared with the currently available radiopharmaceuticals and shows high affinity binding to the vesicular monoamine transporter (VMAT2), which plays an important role in the transfer of monoamines, in particular, neurotransmitters, from cytoplasm into synaptic vesicles. This in turn provides a good correlation between binding of ^18^F-DTBZ analog and innervation of the striatum by dopamine-rich neurons. We substantiated the use of functional imaging modality with classical methods employed to assess the changes in the dopaminergic activity.

In addition to the brain, VMAT2 has been found to be expressed in pancreatic beta cells together with insulin. This co-localization has allowed assessment of beta cell mass by non-invasive imaging using [^11^C]- and [^18^F]-DTBZ in models of diabetes [24]–[26]. One limiting factor, however, for quantifying beta cell mass has been non-specific uptake of radioligand in the pancreas [27]. Recent work has suggested that sigma-1 and sigma-2 receptors not associated with beta cells are responsible for low affinity background binding in pancreas [28]. This and future progress in understanding of radioligand function in pancreas as well as VMAT2 biology should facilitate the application of [^18^F]-DTBZ for PET imaging of disease progression in patients with diabetes.

Our findings demonstrate the considerable loss of dopamine-producing neurons in the mouse striatum as evidenced by the biochemical analysis of different monogenic amines in the brain tissue samples of MPTP treated animals. A significant decrease in the content of dopamine and its metabolic byproducts is observed in the animals that demonstrated characteristic lesions following treatment with MPTP neurotoxin *vs*. control group. In addition, no statistically significant differences were evident in the levels of adrenaline and noradrenaline, which served as control biomarkers in treated and untreated groups. Our results are consistent with previous studies characterizing dopaminergic depletion in mice following treatment with MPTP [29]–[31]. In addition to these findings, tyrosine hydroxylase levels also correlate with neuronal degeneration in the striatum of MPTP treated mice when compared with untreated mice [32], [33].

Non-invasive imaging techniques offer several advantages over the conventional methods, including the longitudinal follow-up of the animals throughout the study without euthanasia and detection of alterations in the neuronal system in presymptomatic animals at an initial stage. We therefore, confirmed our previous results by imaging animals using a PET scanner. We observed reduced uptake of the ^18^F-radiolabeled DTBZ analog in the mouse striatum in the treated *vs.* control groups. Our data is consistent with published reports studying the evolution of dopaminergic reduction in MPTP-induced PD in monkeys [34], and with the clinical studies using different radioactive PET imaging molecules in healthy volunteers [15] and PD patients [35]. Furthermore, the ventral tegmental area (VTA) was found to be weakly stained as compared with the striatum. This difference can be attributed to the fact that dopaminergic neurons present in VTA are less prone to the MPTP-induced lesions.

A major issue with small animals such as mice is the spatial resolution of PET imaging. In PET imaging, the emitted positron travels some distance from the decay before annihilation with an electron resulting in the emission of two 511 KeV gamma rays. The random nature of this travel between the positron and gamma ray points of origin has some impact on the lower limit of spatial resolution obtainable from a scanner. For ^18^F, the travel range of the emitted positron from the decay (E_max_ = 0.633 MeV) is approximately 0.6 mm in water [36]; phantom studies indicated that 1 mm sized objects can be resolved using ^18^F labeled tracers and appropriate scanner technology. Another factor is the partial volume effect, which must be considered when performing manual analysis (e.g., identifying the regions of interest). However, data had the advantage of registered CT data with a resolution of 0.1 mm^3^. This high-resolution CT data allowed the PET data to be registered (and resliced) to a standard atlas to identify the brain structures of interest. Thus, in our analysis the volume of the sampled area was almost 10 mm^3^ in each image set, which should minimize any potential partial volume effects on the quantitative results.

In conclusion, our approach should provide the basis to use ^18^F-labeled DTBZ analogs as potent radiotracers to follow up the process of PD involving dopamine dysfunction by positron emission tomography. This may help in the advent of novel diagnostic and therapeutic agents required for both the early assessment and treatment of this neurodegenerative disorder.
